# Assessment of vestibulo-ocular reflex and its adaptation during stop-and-go car rides in motion sickness susceptible passengers

**DOI:** 10.1007/s00221-023-06619-4

**Published:** 2023-04-25

**Authors:** Cecilia Ramaioli, Tobias Steinmetzer, Adrian Brietzke, Paul Meyer, Rebecca Pham Xuan, Erich Schneider, Martin Gorges

**Affiliations:** 1 Institute of Medical Technology, Brandenburg University of Technology Cottbus-Senftenberg, Cottbus, Germany; 2 Ergonomics and Innovation, Chemnitz University of Technology, Chemnitz, Germany; 3 Group Innovation, Volkswagen AG, Wolfsburg, Germany; 4 Fraunhofer Institute for Ceramic Technologies and Systems, Dresden, Germany

**Keywords:** Video head impulse test, Vestibulo-ocular reflex, Adaptation, Motion sickness, Stop-and-go car ride

## Abstract

Motion sickness is a physiological condition that negatively impacts a person's comfort and will be an emerging condition in autonomous vehicles without proper countermeasures. The vestibular system plays a key role in the origin of motion sickness. Understanding the susceptibility and (mal) adaptive mechanisms of the highly integrated vestibular system is a prerequisite for the development of countermeasures. We hypothesize a differential association between motion sickness and vestibular function in healthy individuals with and without susceptibility for motion sickness. We quantified vestibular function by measuring the high-frequency vestibulo-ocular reflex (VOR) using video head impulse testing (vHIT) in 17 healthy volunteers before and after a 11 min motion sickness-inducing naturalistic stop-and-go car ride on a test track (Dekra Test Oval, Klettwitz, Germany). The cohort was classified as motion sickness susceptible (*n* = 11) and non-susceptible (*n* = 6). Six (out of 11) susceptible participants developed nausea symptoms, while a total of nine participants were free of these symptoms. The VOR gain (1) did not differ significantly between participant groups with (*n* = 8) and without motion sickness symptoms (*n* = 9), (2) did not differ significantly in the factor time before and after the car ride, and showed no interaction between symptom groups and time, as indicated by a repeated measures ANOVA (*F*(1,15) = 2.19, *p* = 0.16. Bayesian inference confirmed that there was “anecdotal evidence” for equality of gain rather than difference across groups and time (BF_10_ < 0.77). Our results suggest that individual differences in VOR measures or adaptation to motion sickness provocative stimuli during naturalistic stop-and-go driving cannot predict motion sickness susceptibility or the likelihood of developing motion sickness.

## Introduction

Motion sickness is a complex syndrome (Lackner 2014) characterized by various signs and symptoms, including discomfort, nausea, and vomiting, that can manifest in healthy individuals due to continuous passive self-motion (Bertolini and Straumann 2016). This uncomfortable state can occur in any transportation system (e.g., ship (Irwin 1881), bus (Irwin 1881; Turner and Griffin 1999)) and is becoming increasingly relevant in automated vehicles (Diels et al. 2016). Self-driving vehicles as characterized by the International Society of Automotive Engineers (SAE International 2014), provide an opportunity for former drivers to be relieved of responsibilities, such as steering the vehicle or monitoring the environment. Customers want to use their travel time to eat, sleep, or watch movies (Kyriakidis et al. 2015), but the user group of potential early adopters of self-driving vehicles has an increased likelihood of experiencing common symptoms of motion sickness (Brietzke et al. 2022), especially when considering some of the desired activities. Detailed knowledge of the physiology underlying motion sickness is a prerequisite for defining effective non-pharmacological countermeasures against motion sickness. Such countermeasures can be designed as a model-based motion control system of the transportation system (Braccesi and Cianetti 2011). Motion sickness is a well-known physiological condition (Irwin 1881) even though the underlying neurobiology is still not fully understood.

A well-known theory underlying the development of motion sickness is based on conflicts of sensory information between different sensory modalities, including visual-vestibular sensory conflicts (Held 1961). More recent theories also consider a possible unimodal mismatch between observed sensory signals and the expectation of sensory input derived from internal models. Since the mismatch may result from an error in either the expectation or the sensory input, the system could rely more on one or the other (Nooij et al. 2021). Some individuals may habituate to a persistent error prediction rather than minimize the error through adaptation. Motion sickness resulting from a mismatch between the sensory input and the internal model may be explained by a lack of adaptation or habituation. Conversely, persistent motion sickness may also affect VOR performance, as suggested by the study of Idoux et al. (2018). For example, the internal model is continuously updated, so that adaptation to a provocative motion exposure (e.g., from a boat ride, roller coaster) can lead to a condition called “land sickness” after the motion exposure ends (Golding 2016). In addition, adaptation can reduce low-frequency yaw VOR gain in response to a visuo-vestibular mismatch resulting from exposure to open-sea conditions (Kolev and Tibbling 1992), which mainly cause heave, pitch and roll, but not yaw motion (Wertheim et al. 1998). Importantly, even short exposures lasting only a few minutes are well able to induce adaptation of the high-frequency VOR (Migliaccio and Schubert 2013).

The vestibular system plays a key role in the mechanism that causes motion sickness, because it provides the primary actual sensory information about the forces acting on the head (Cullen 2019). The video-based head impulse test (vHIT) provides an objective measure of vestibular function (Halmagyi et al. 2017). The primary output of the vHIT is the vestibulo-ocular reflex (VOR) gain, which is the ratio of eye and head velocity (Gordon et al. 1996). VOR gain and its left–right asymmetry appear to be related to individual susceptibility to motion sickness in humans (Neupane et al. 2018). In mice, lower VOR gain has been reported as a protective non-pharmacological mechanism to minimize motion sickness (Idoux et al. 2018). In contrast, opioid-induced abnormally low VOR gain values (~ 0.6) in humans have been identified as highly correlated with the development of severe motion sickness when exposed to head motion (Lehnen et al. 2015). Exposure to discordant visual-vestibular stimuli can lead to changes in VOR gain on a physiological scale, which can occur on different time scales (Colagiorgio et al. 2015).

We hypothesize a differential association between motion sickness and vestibular function in healthy individuals with and without motion sickness susceptibility. Specifically, we hypothesize that VOR gain, as a measure of vestibular function, will change after a provocative car-ride depending on whether participants develop nausea symptoms.

## Materials and methods

### Participants

A total of 20 healthy volunteers (mean age 37.4 years (standard deviation (SD) 13.8), range 19–62 years, 11 males) participated in the study after providing written and informed consent. The study was approved by the Ethics Committee of the Brandenburg University of Technology, Cottbus, Germany (EK2018 7) and was conducted according to the tenets of the Declaration of Helsinki, 1975, as revised and valid at the time of the study.

To estimate the required number of study participants, we performed a sample size calculation for a two factorial repeated measures ANOVA with interaction between group and time using G*Power (version 3.1.9.6) (Faul et al. 2007). For all power calculations, we used typical values for significance level (*α* = 0.05) and the power (1-*β* = 0.8). We assumed a within-group difference in VOR gain for individuals with motion sickness symptoms of 0.1, while assuming no difference in VOR gain for asymptomatic individuals. Using a within group SD explained by effect of 0.05, a correlation between repeated measures of 0.7 (Lehnen et al. 2015), and two groups and two repeated measures, the power analysis for a repeated measures ANOVA with interaction yields a total sample size of 15 participants. Consistent with these assumptions, the results of the present study are almost identical to the assumptions for the power analysis.

We enrolled 20 participants, of which three participants with unacceptable data quality were excluded. The remaining 17 participants provided adequate power for our study. All participants had a valid driver's license and reported no history of cardiovascular, neurological, or vestibular problems. None of the participants had clinically significant medical conditions or vestibular disorders.

### Experimental procedure

Participants were directly asked to rate how often they experience motion sickness while driving, using the options “never”, “rarely”, “occasionally”, “frequently”, and “almost always”.

In addition, motion sickness susceptibility was assessed using the German version of the Motion Sickness Susceptibility Questionnaire, MSSQ-Short (Golding 2006). The MSSQ-Short assesses self-reported motion sickness experiences in childhood and adulthood for different types of motion on a 4-point rating scale ranging from zero (never felt sick) to three (frequently felt sick). The weighted score of the MSSQ-Short is a quantitative predictor of individual differences in motion sickness susceptibility.

**Fig. 1:**
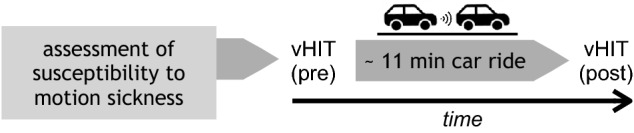
Experimental procedure. Prior to the recording sessions, all participants were classified to either a "susceptible" or "non-susceptible" to motion sickness. All participants underwent a video head impulse test (vHIT) before and after the stop-and-go car ride

The experimental design is shown in Fig. 1. During each car ride, a psychophysical assessment of nausea was performed every minute to quantify the symptoms of nausea on an 11-point integer scale (zero, "no occurrence", to ten, "unbearable").

### Vestibular testing

All participants underwent a standard monocular video head impulse test using the EyeSeeCam® (EyeSeeTec GmbH, Munich, Germany) to assess horizontal rotational VOR function (Bartl et al. 2009). The EyeSeeCam® was operated at 220 Hz sampling rate and provided synchronized eye and head movement recordings. Vestibular testing was performed by an experienced experimenter (C.R.) who stood behind the subject and passively rotated the subject's head around the earth vertical axis within a small amplitude range of up to ± 20°, allowing the head to reach high angular accelerations (up to 4700°/s^2^). The participant was asked to fixate a small, fixed target dot (eye-to-target distance approximately 2 m). To avoid anticipation, the abrupt head impulses were randomized in direction (left and right) and timing.

### vHIT data analysis

The built-in EyeSeeCam® software was used to detect and separate the data into individual head impulses. The software provides head and eye velocity traces over time for each head impulse. The VOR gain for each head impulse was calculated as the ratio of the mean eye velocity to the mean head velocity for a 10 ms time window centered at 60 ms after head impulse onset. To assess VOR gain asymmetry, an asymmetry index (Schmid-Priscoveanu et al. 2001) was calculated as the ratio between the difference and the sum of leftward and rightward VOR gains expressed as a percentage. Head impulses were considered valid if the peak head velocity exceeded 100°/s. A minimum of five valid impulses in each direction was required for further data analysis. The total VOR gain for each participant and direction was calculated by averaging the VOR gains for the five fastest valid head impulses (with respect to peak head velocity).

### Car ride

All participants underwent a car ride on a test track (Dekra Test Oval, Klettwitz, Germany) as a passenger in the front seat. The passenger was restrained with a three-point seat belt with no additional head stabilization beyond the standard head restraint to simulate a normal car ride. Two left-hand drive cars (Volkswagen Passat, B8) were driven by certified test drivers. The car ride lasted approximately 11 min with a predefined and realistic stop-and-go motion profile. The motion profile consisted of two alternating low-intensity and high-intensity acceleration episodes along the longitudinal direction. A preceding vehicle was dynamically controlled by a predefined velocity-over-time trajectory. The second vehicle, carrying the participant, was controlled by adaptive cruise control. This setup ensured a reproducible stimulation resulting in a mean velocity of 3.32 m/s (maximum positive acceleration of 2.19 m/s^2^, maximum negative acceleration of -2.28 m/s^2^). The resulting stimulation had a peak acceleration frequency in the range of 0.07 Hz. During the car ride, participants were asked to focus on a movie of a short documentary shown on a 10-inch screen. The screen was mounted on the dashboard of the car at a height of about 30 cm below eye level with a horizontal distance of about 70 cm. At the end of the ride, two questions related to the documentary had to be answered to keep the participant focused. For more details on the experimental setup, see Brietzke et al. (2021a, b).

### Assessment of motion sickness

Every minute during the drive, participants received a psychophysical assessment of nausea symptoms and a verbal rating of nausea status, which was quantitatively recorded on an 11-point integer scale ranging from zero (no nausea, “all right”) to ten (“intolerable”) (Apfel et al. 2004). For ethical reasons, experimenters were instructed to terminate the car ride if participants scored above seven on the nausea scale, if participants showed signs of severe malaise, or if participants requested termination of the session.

### Statistical data analysis

Continuous data are presented as mean (SD), categorical data as absolute numbers or percent. Statistical calculations were performed using JASP (JASP Team 2022) and Python (version 3.9). Pandas (version 1.3.2) (McKinney et al. 2011) and SciPy (version 1.7.1) (Virtanen et al. 2020) were used. Differences in VOR gain between groups and time were assessed using repeated measures analysis of variance (ANOVA). The within factor was time (before and after the car ride). For repeated measures ANOVA, both frequentist and Bayesian analyses were performed. Spearman rank order correlations were used to examine possible correlations. *P* < 0.05 was considered statistically significant for frequentist analysis. All tests were two-sided.

## Results

### Motion sickness susceptibility and nausea

None of the 20 participants had the car ride aborted due to any of the above criteria (e.g., severe nausea). Despite possible symptoms of motion sickness, all participants were able and willing to complete all vHIT measurements. Data from three participants (out of 20) were excluded due to an insufficient number of valid head impulses with acceptable data quality.

**Fig. 2:**
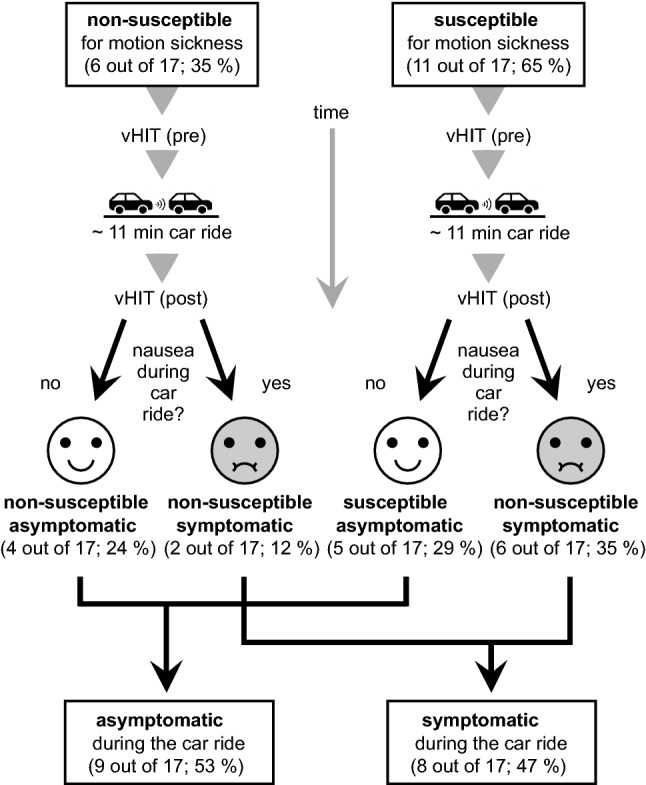
Seventeen participants were divided into four subgroups based on self-reported susceptibility and nausea outcome during motion exposure. Participants were divided into two groups, "asymptomatic" and "symptomatic", based on the occurrence of nausea symptoms (nausea score greater than zero) during the car ride

These 17 subjects were classified according to the described pre-experimental self-report of general motion sickness susceptibility according to Brietzke et al. (2021a) as follows: Six participants answered “never” and were classified as non-susceptible to motion sickness, while 11 participants answered other than “never” and were classified as susceptible. From the total cohort, eight (out of 17, 47%) experienced nausea symptoms, while the remaining nine (out of 17, 53%), including both non-susceptible and susceptible participants, did not experience nausea symptoms. This allows the definition of the four subgroups shown in Table 1 and Fig. 2.

**Table 1:** Demographic features and VOR gains for all individuals

	Non-susceptible	Susceptible	Analyzed groups
Symptomatic			
Count	*n* = 2	*n* = 6	*n* = 8
Age/years	46.5 (6.4) [42–51]	33.5 (10.5) [20–45]	36.8 (11.0) [20–51]
MSSQ-Short	10.1 (14.3) [0.0–20.3]	19.7 (9.3) [7.6–28.9]	17.3 (10.5) [0.0–28.9]
Nausea scale	1.0 (0.0) [1–1]	2.7 (0.8) [2–4]	2,8 (0.7) [1–4]
Pre VOR gain	1.03 (0.07) [0.98–1.08]	1.04 (0.03) [0.99–1.08]	1.04 (0.04) [0.98–1.08]
Post VOR gain	1.03 (0.04) [1.00–1.06]	1.03 (0.04) [0.97–1.08]	1.03 (0.04) [0.97–1.08]
Asymptomatic			
Count	*n* = 4	*n* = 5	*n* = 9
Age/years	35.8 (19.2) [21–62]	33.6 (17.8) [19–54]	34.6 (17.2) [19–62]
MSSQ-Short	2.7 (3.7) [0.0–7.9]	10.5 (2.8) [7.3–13.4]	7.1 (5.1) [0.0–13.4]
Nausea scale	0 (0) [0–0]	0 (0) [0–0]	0 (0) [0–0]
Pre VOR gain	1.04 (0.03) [1.00–1.08]	1.01 (0.09) [0.94–1.16]	1.02( 0.07) [0.94–1.16]
Post VOR gain	1.03 (0.03) [0.99–1.06]	1.02 (0.10) [0.96–1.19]	1.03 (0.07) [0.96–1.19]

Values are provided as mean (SD) [min, max]

*MSSQ* Motion sickness susceptibility questionnaire. *VOR* vestibular ocular reflex

As shown in Table 1, most non-susceptible participants had no nausea symptoms and most susceptible participants had nausea symptoms. This means that there is no identity between the susceptibility group and the occurrence of nausea symptoms (nausea score greater than zero) during the car ride. However, there is a significant correlation between the previously reported susceptibility (MSSQ-Short score) and the nausea score (*r* = 0.53, *p* = 0.028). For the following statistical analysis, the presence of nausea symptoms (nausea score greater than zero) is used as a between-subjects factor, resulting in two groups, i.e., "symptomatic" and "asymptomatic", as shown in Fig. 2.

### Vestibular function

**Fig. 3:**
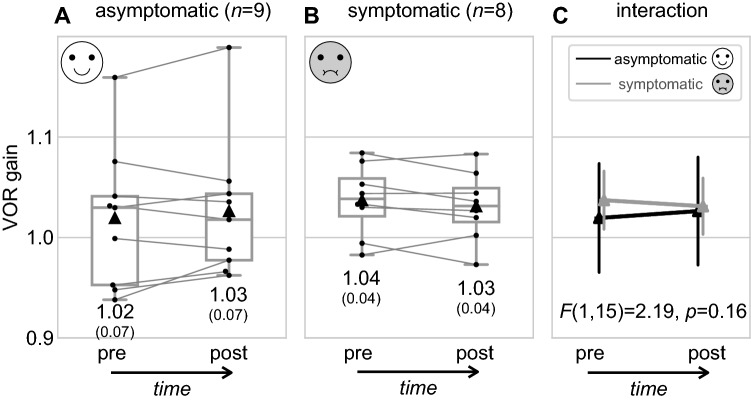
Boxplots showing groupwise VOR gain values before (pre) and after (post) the car ride for asymptomatic participants (**A**) compared to symptomatic participants (**B**). Dots represent individual VOR gain values connected by a gray line for each participant. Black triangles represent mean VOR gain values for groups and timepoints. **C** Interactions between pre-post measures and symptomatic groups, i.e., symptomatic (gray) and asymptomatic (black)

Subjects experienced a mean of 9.6 (SD 2.4) valid head impulses to the left and a mean of 10.0 (SD 3.0) to the right. There was no significant difference in direction, i.e., left versus right head impulses, (*F*(1,33) = 0.619, *p* = 0.437) for the individual VOR gain values. Therefore, left and right VOR gains were pooled. Figure 3 summarizes the VOR gain results.

Next, we investigated whether the VOR gain before (pre) and after (post) motion provocation was predictive of nausea symptoms (Table 1). For the following statistical analysis, the groups "symptomatic" and "asymptomatic" were used as a categorical between-subjects factor (Table 1, last column). No statistically significant effect was found for the within-subject comparison (pre–post) (*F*(1,15) = 0.03, *p* = 0.86) and between-subject group comparison (*F*(1,15) = 0.17, *p* = 0.69). Overall, there was no significant effect between groups or pre–post VOR gains. Bayesian analysis supported this notion as indicated by Bayesian repeated measures ANOVA (Wagenmakers et al. 2018). Importantly, no significant interaction between pre–post measures and symptomatic groups was observed (*F*(1,15) = 2.19, *p* = 0.16), as shown in Fig. 3C. The Bayesian factor for any effect or interaction in the repeated measures ANOVA model was BF_10_ < 0.77, indicating "anecdotal evidence" of equality rather than difference (Quintana and Williams 2018).

The occurrence of nausea symptoms during the car ride had no significant effect on VOR gain asymmetry (*F*(1,15) = 0.311, *p* = 0.586). Pooling across pre and post conditions yielded mean asymmetry values of 3.02% (SD 2.49%) and 3.28% (SD 2.54%) in the asymptomatic and symptomatic groups, respectively. In the pre timepoint, we observed comparable average asymmetries of 2.83% (SD 2.88%) and 3.59% (SD 3.26%), respectively. In summary, there were no differences between the VOR metrics across groups and time.

## Discussion

During the car ride, we were able to induce symptoms of nausea in some participants. Participants with symptoms were not exclusively from the motion sickness susceptible group, but also from the non-susceptible group. Using vHIT-based vestibular function testing before and after a standardized car ride on a test track in motion sickness susceptible and non-susceptible participants, we found no difference in the high-frequency VOR at any timepoint. In the present study, some participants developed mild to moderate symptoms of motion sickness within approximately 11 min, but VOR gain values were unaffected by the provocative car ride. The present results are consistent with a previous study by Dai et al. (2011), who reported that susceptible and non-susceptible participants maintained their low-frequency VOR gains during exposure to motion sickness provocative stimuli.

Nevertheless, a decrease in VOR gain over time in response to a visuo-vestibular mismatch, as reported in the context of habituation to sea conditions (Kolev and Tibbling 1992), leads to an increased threshold for the development of motion sickness (Shupak et al. 1990). The same phenomenon has been demonstrated in professional figure skaters (Tanguy et al. 2008) and ballet dancers (Nigmatullina et al. 2015), who showed reduced VOR gain after specific training. As a possible consequence, figure skaters and ballet dancers may be less susceptible to motion sickness, but the time period for which the VOR gain appears to be "adapted" remains to be determined. A moderately reduced VOR gain may be a protective mechanism to prevent the later onset of motion sickness, as demonstrated in a mouse model (Idoux et al. 2018). A recent study (Neupane et al. 2018) is generally consistent with this body of evidence and shows subtle differences in high frequency VOR gain between susceptible and non-susceptible individuals. However, a significantly reduced VOR gain in healthy individuals (e.g., VOR gain of about 0.6 after opioid administration) is associated with a very high risk of motion sickness (Lehnen et al. 2015).

These reports of reduced VOR gain in the literature raise the question of why we did not observe an effect on VOR gain before and after exposure to motion stimuli. The present study assessed susceptibility to motion sickness, whereas most other studies have focused on motion sickness (Kolev and Tibbling 1992; Shupak et al. 1990; Nachum et al. 2002), “artificial” motion stimuli (Idoux et al. 2018), rotating motion stimuli in the low frequency range (~ 0.01 Hz) (Dai et al. 2011), caloric stimulation (Kolev and Tibbling 1992), or assessing pure motion sickness susceptibility based on a retrospective questionnaire (e.g., MSSQ-Short) without exposing study participants to motion stimuli (Neupane et al. 2018). Participants in our study may not have been habituated to a provocative motion sickness stimulus for a sufficiently long period of time, such as professional skaters or dancers (Tanguy et al. 2008; Nigmatullina et al. 2015), for whom the ability to adapt their VOR gain may be a personal trait rather than a general mechanism. Therefore, we can hypothesize that none of our study participants had relevant "protection" (adaptation) against motion provocative stimuli. Different transportation systems stimulate the vestibular system in different ways, and the duration of stimulation varies considerably (from minutes in a car to days at sea).

Similar to the results of Yang et al. (2016), we demonstrated a VOR gain asymmetry of approximately 3% both before and after stimulation, regardless of susceptibility to motion sickness or development of nausea (no difference between groups). These results contrast with a recent study with a similar study design by Neupane et al. 2018, who reported pronounced asymmetries on the order of 10%. Interestingly, the standard deviation in their susceptible group, but not in their non-susceptible group, was also more than three times greater than in our symptomatic group and, more importantly, it was also greater than the normative range of 5.6% previously reported for gain asymmetries (Schmid-Priscoveanu et al. 2001). These discrepancies need to be addressed in future studies.

We assessed vestibular function using the vHIT, which tests the horizontal VOR in the range of physiological frequencies (5–7 Hz (Carriot et al. 2017)). The VOR response is determined by (1) the high-pass properties of the peripheral semicircular canal, which explains the sub-unity VOR gain at low frequencies (Braccesi and Cianetti 2011 and Dai et al. 2011), and (2) the central velocity storage mechanism, which prolongs the high-pass time constant of the VOR and provides an explanation for the adaptation of low-frequency VOR gain (Cohen et al. 2003; Laurens and Angelaki 2011). In contrast, the high-frequency vHIT is not affected by either the high-pass properties of the semicircular canal or by velocity storage. Our observation of similar high-frequency vHIT gains in susceptible and non-susceptible participants supports the hypothesis that the VOR gain differences previously observed with low-frequency stimulation appear to be caused exclusively by differences in group-dependent velocity storage time constants (Cohen et al. 2003; Laurens and Droulez 2007; Laurens and Angelaki 2011; 2017). This implies that in the non-physiological lower frequency range, VOR gain and time constant are directly and positively related (Tanguy et al. 2008), allowing elevated values of one or both of these parameters to be used as predictors of susceptibility to motion sickness. In contrast, higher VOR gain in the physiological frequency range is not a sufficient predictor of motion sickness. A simple conclusion is that the type of motion stimulus (head motion profile in all 6 degrees of freedom) together with the functional integration of the vestibular system including the internal model plays a key role in understanding the development of motion sickness.

The standardized stop-and-go profile was designed to expose the car to linear acceleration rather than angular acceleration. The study participants were restrained with a three-point seat belt with no additional head stabilization other than the standard head restraint. As a result, the applied linear force during braking or acceleration caused primarily head pitch, depending on the head position. Head pitch is a rotation around the interaural axis, but we tested the VOR elicited by a rotation of the head around the yaw axis. Both types of head rotation evoke an angular VOR, from which we hypothesized that the vestibular sensory system in general is stimulated. We were unable to provide evidence for this hypothesis, so the discrepancy in the direction of VOR stimulation must be considered a limitation of the present study. In addition, the effect of linear acceleration acting on the otoliths remains open and should be investigated in future studies. This study is also limited by the relatively small number of participants, although the power analysis showed that the study was adequately powered. Furthermore, the proportion of participants who developed symptoms of nausea during the car ride was relatively low. In addition, the reported nausea symptoms were only mild to moderate, as indicated by a maximum nausea score of 4 (out of 10). Although the motion profiles were designed to be as realistic as possible, driving conditions on a test track remain artificial. The motion profile did not induce severe nausea symptoms (e.g., vomiting), but was below the desired nausea outcome. In addition, the motion sickness provocative stimulation time in this study of about 11 min may have been too short to induce a measurable adaptation of the VOR gain.

To address some of these limitations, future studies should include more participants with a higher risk of developing severe motion sickness symptoms to cover a broader range of nausea symptoms. In addition, the velocity storage time constant should also be considered. The velocity storage time constant is associated with motion sickness (Hoffer et al. 2003) and can be influenced by habituation, which in turn reduces the risk of motion sickness (Dai et al. 2011; Cohen et al. 2008). Future studies should use the vHIT as a useful screening tool for subjects prior to enrolment. A VOR gain of less than 0.75 (Mossman et al. 2015) would identify asymptomatic vestibular deficits, which have a prevalence of 2.4% in the population younger than 48 years (Grill et al. 2018). Participants with such a deficit should, therefore, be excluded from studies on motion sickness to avoid potential confounding. In addition, a head-mounted eye tracking device with a scene camera (Marx et al. 2012) could be used to study free viewing during the presentation of provocative motion stimuli. These video data could provide further insights into the relationship between head motion, visual exploration behavior, and motion sickness symptoms.

In summary, we show that a stop-and-go car ride can induce motion sickness in passengers, but does not have a direct effect on the high-frequency VOR gain within a relatively short period of time. VOR gain is also not associated with susceptibility to motion sickness, regardless of whether passengers experience motion sickness or not. These findings are important for the field of motion sickness research in the context of self-driving vehicles. We investigated the well-established vHIT as a complement to state-of-the-art driver assistance technologies. Our results, which provide anecdotal evidence of equality rather than difference of VOR functionality between participants with and without motion sickness symptoms, have the potential to spark a controversial scientific discussion about whether the vestibular systems in the motion sickness susceptible population differ from controls. Advanced models of the vestibular system could both improve our understanding of motion sickness susceptibility and provide an essential basis for the development of effective non-pharmacological countermeasures to ultimately prevent motion sickness in passengers.

## Data Availability

Data are available upon reasonable request.
